# Recognizing the Differentiation Degree of Human Induced Pluripotent Stem Cell-Derived Retinal Pigment Epithelium Cells Using Machine Learning and Deep Learning-Based Approaches

**DOI:** 10.3390/cells12020211

**Published:** 2023-01-04

**Authors:** Chung-Yueh Lien, Tseng-Tse Chen, En-Tung Tsai, Yu-Jer Hsiao, Ni Lee, Chong-En Gao, Yi-Ping Yang, Shih-Jen Chen, Aliaksandr A. Yarmishyn, De-Kuang Hwang, Shih-Jie Chou, Woei-Chyn Chu, Shih-Hwa Chiou, Yueh Chien

**Affiliations:** 1 Department of Information Management, National Taipei University of Nursing and Health Sciences, Taipei 112303, Taiwan; 2 Department of Biomedical Engineering, National Yang Ming Chiao Tung University, Taipei 112304, Taiwan; 3 Department of Medical Research, Taipei Veterans General Hospital, Taipei 112201, Taiwan; 4 School of Medicine, National Yang Ming Chiao Tung University, Taipei 11217, Taiwan; 5 Institute of Anatomy and Cell Biology, National Yang Ming Chiao Tung University, Taipei 112304, Taiwan; 6 Institute of Clinical Medicine, National Yang Ming Chiao Tung University, Taipei 112304, Taiwan; 7 Institute of Food Safety and Health Risk Assessment, School of Pharmaceutical Sciences, National Yang Ming Chiao Tung University, Taipei 112304, Taiwan; 8 Department of Ophthalmology, Taipei Veterans General Hospital, Taipei 11217, Taiwan; 9 Institute of Pharmacology, School of Medicine, National Yang Ming Chiao Tung University, Taipei 112304, Taiwan

**Keywords:** induced pluripotent stem cells, retinal pigment epithelial cells, artificial intelligence, deep learning, convolutional neural network, traditional machine learning

## Abstract

Induced pluripotent stem cells (iPSCs) can be differentiated into mesenchymal stem cells (iPSC-MSCs), retinal ganglion cells (iPSC-RGCs), and retinal pigmental epithelium cells (iPSC-RPEs) to meet the demand of regeneration medicine. Since the production of iPSCs and iPSC-derived cell lineages generally requires massive and time-consuming laboratory work, artificial intelligence (AI)-assisted approach that can facilitate the cell classification and recognize the cell differentiation degree is of critical demand. In this study, we propose the multi-slice tensor model, a modified convolutional neural network (CNN) designed to classify iPSC-derived cells and evaluate the differentiation efficiency of iPSC-RPEs. We removed the fully connected layers and projected the features using principle component analysis (PCA), and subsequently classified iPSC-RPEs according to various differentiation degree. With the assistance of the support vector machine (SVM), this model further showed capabilities to classify iPSCs, iPSC-MSCs, iPSC-RPEs, and iPSC-RGCs with an accuracy of 97.8%. In addition, the proposed model accurately recognized the differentiation of iPSC-RPEs and showed the potential to identify the candidate cells with ideal features and simultaneously exclude cells with immature/abnormal phenotypes. This rapid screening/classification system may facilitate the translation of iPSC-based technologies into clinical uses, such as cell transplantation therapy.

## 1. Introduction

Induced pluripotent stem cell (iPSC) technologies have been shown to hold great promise for personalized therapy, translation medicine, and regeneration medicine [1]. Owing to the potential of iPSCs to be generated from somatic cells via the transduction of specific transcription factors [2], scientists reprogrammed patient-derived somatic cells to generate patient-specific iPSCs [3,4,5]. Remarkably, silencing the expression of human leukocyte antigen (HLA) class I allows to establish the universal human leukocyte antigen (HLA) iPSCs characterized by low immunogenicity [6]. In addition, following the stimuli by defined factors, iPSCs can undergo differentiation into different lineages and exhibit robust phenotypic and genotypic changes [2,7]. For instance, iPSCs can be differentiated into various retinal cell types, including retinal pigment epithelial cells (iPSC-RPEs) [8], retinal ganglion cells (iPSC-RGCs) [9,10], and retinal organoids [11,12]. These iPSC-derived retinal cell lineages have been utilized for disease modeling [9], pathogenesis investigation [9], and regeneration medicine [13].

Age-related macular degeneration (AMD), including dry and wet types, is the leading cause of visual impairment or severe visual loss in the elderly population in developing countries [14]. In a study by Takahashi et al., patient-derived skin fibroblasts were used to generate iPSC-RPEs for transplantation [15]. These iPSC-RPEs exhibited high quality, consistency, safety, and could be generated in large quantities [16]. The generation of iPSC-derived cell products for clinical application requires complicated characterization and quality control, which is a very time-consuming and labor-intensive work [15,16]. These disadvantages may limit the productive efficiency and sufficient quantity of iPSC-RPEs, therefore reducing the clinical applicability of iPSC-derived cell products.

Deep learning is as a branch of machine learning that constitutes the field of artificial intelligence (AI) [17]. Traditional machine learning algorithms require extracting the features from the original data through feature engineering and have been widely used for image recognition [18,19]. However, these algorithms are predominantly designed to deal with low-dimensional data and therefore carry some disadvantages [20]. The technologies of deep learning originated from the artificial neural networks (ANNs) that can achieve learning goals via the acquisition of large data [21]. Unlike traditional machine learning, which processes the original images by splicing or extracting their features, deep learning algorithms are able to extract the features automatically by convolving the original images, followed by pooling, downsampling, or upsampling [22]. There are various widely used traditional machine learning algorithms, including the support vector machines (SVMs), random forests (RFs), and principal component analysis (PCA). On the contrary, frequently used deep learning algorithms include multilayer perceptrons (MLPs), recurrent neural networks (RNNs), and convolutional neural networks (CNNs). Owing to their multiple advantages, deep learning algorithms have been extensively used in image analysis and other biomedical applications [23].

CNNs represent one of the deep learning algorithms. Several studies reported the use of CNNs for cell classification and the evaluation of cell differentiation. For example, Niioka et al., used CNNs to identify the stage of C2C12 mouse myoblast differentiation with an accuracy rate of 91.3%. After data augmentation by flipping and rotation, the accuracy rate of evaluation was improved by 10% [24]. Kusumoto et al., used CNN to distinguish iPSC-derived endothelial cells and non-endothelial cells with an accuracy rate of more than 90% [25]. Orita et al., employed CNN to examine the readiness of cultivated iPSC-derived cardiomyocytes for experimental procedures with an accuracy of 89.7% [26]. Overall, these reports demonstrated that CNN could be used as a feasible tool for biological image recognition and the evaluation of cell maturation and differentiation. To meet the demands of iPSC technologies in personalized therapy, translational medicine, and transplantation, the cultivation, maintenance, and the characterization of differentiated cells require large-scale manpower and management efforts. In this study, we aimed to establish an AI-based screening system that can rapidly screen the iPSC-derived cell lineages and recognize the differentiated cells with ideal morphologies and other features, while simultaneously excluding the cells with immature or other unexpected phenotypes. This rapid screening/selection system may facilitate the translation of iPSC-based technologies into clinical uses, such as cell transplantation therapy.

## 2. Materials and Methods

### 2.1. Cell Culture

iPSCs were seeded on the dishes pre-coated with Geltrex™ matrix (#A1413301; Thermo Fisher Scientific, Waltham, MA, USA) dissolved in DMEM/F-12 (#10565-018; Thermo Fisher Scientific) and maintained in StemFlex™ medium (#A3349401; Thermo Fisher Scientific, Waltham, MA, USA) in a humidified incubator at 37 °C and 5% CO_2_. iPSCs were subcultured by dissociation in Versene solution (#15040066; Thermo Fisher Scientific, Waltham, MA, USA) and re-seeding in StemFlex medium. The induction of iPSC-MSC differentiation was conducted following the methods described by Hynes et al. [27]. Briefly, undifferentiated iPSCs without mouse embryonic fibroblast feeders were dissociated with a cell scraper and washed using the MSC wash medium containing MEM α-based medium supplemented with 5% fetal bovine serum, 50 U/mL penicillin, and 50 μg/mL streptomycin. The wash medium with feeder-free iPSC suspension was centrifuged at 400× *g* for 5 min at 4 °C, and then the supernatant was removed. Subsequently, these iPSC colonies were resuspended in MSC culture medium consisting of MEM α-based medium supplemented with 10% fetal bovine serum, 2 mM L-glutamine, 1 mM sodium pyruvate, 50 U/mL penicillin, and 50 μg/mL streptomycin, non-essential amino acids (NEAA), and HEPES solution. The medium was changed every 3 to 4 days, and iPSC colonies were differentiated into a mixture of heterogenous cell types after a 2-week differentiation time course. The serial passaging was used to select MSC-like cells which were more homogenous and exhibited pronounced fibroblast morphology. For the differentiation of iPSCs into iPSC-RPEs and iPSC-RGCs, the differentiation following the procedures reported by Regent et al. [28], Yang et al. [8] and the protocols for the routine generation of iPSC-RGCs in our laboratory [9,10] were used, respectively.

### 2.2. Image Acquisition and Image Data Augmentation

**Figure 1:**
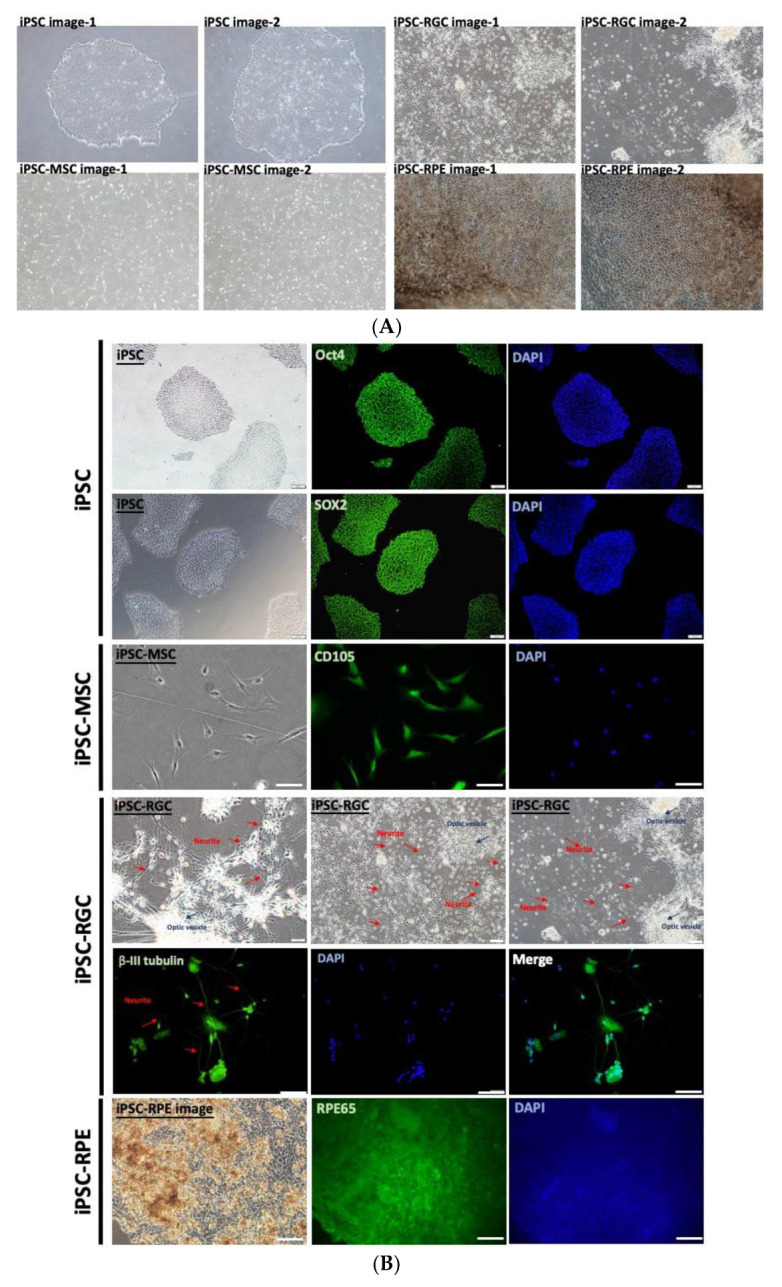
Characterization of iPSCs, iPSC-MSCs, iPSC-RGCs, and iPSC-RPEs with optimal differentiation. (**A**) Representative images of iPSCs, iPSC-MSCs, iPSC-RGCs, and iPSC-RPEs obtained by phase-contrast microscopy. (**B**) Validation of the indicated cell types by immunofluorescent staining of the indicated lineage-specific markers. Nuclei stained with DAPI. Scale bars = 100 μm.

To establish the AI-based iPSC screening system that can rapidly recognize iPSCs and iPSC-derived cell lineages, we sought to use both traditional machine learning and deep learning algorithms to recognize the images of iPSCs and iPSC-derived retinal or mesenchymal lineage cells. Meanwhile, we attempted to use these algorithms to evaluate the differentiation degree of iPSC-derived RPEs. All the images of iPSCs and iPSC-derived lineage cells were captured by qualified technicians or research assistants using the inverted fluorescence microscope (Olympus IX71; Olympus Corporation, Tokyo, Japan). At the end of differentiation procedures, all differentiated lineages cells (i.e., iPSC-MSCs, iPSC-RGCs, and iPSC-RPEs) with any given differentiation degree were enrolled in this study and cell images were obtained. Representative images of undifferentiated iPSCs and iPSC-derived cells with optimal differentiation are shown in Figure 1A. Under the cultivation conditions using the aforementioned protocols, undifferentiated iPSCs consistently formed compacted colonies exhibiting well-defined margins, large nuclei, less cytoplasm, and other embryonic stem cell-like morphologies (Figure 1A), consistently with previously published results [29]. After receiving the stimuli and undergoing proper differentiation, iPSC-MSCs presented typical stromal cell- or fibroblast-like morphologies (Figure 1A), which were apparently different from those of undifferentiated iPSCs. Following the stepwise differentiation protocol [10] for iPSC-RGCs, iPSCs formed neuroepithelium and optic vesicles at days 16 and 46 post-induction, respectively. Eventually, the optic vesicles were switched into neuronal culture with B-27 supplement and Notch signaling inhibitor to differentiate into iPSC-RGCs (Figure 1A). For the differentiation of iPSC-RPEs, several remarkable pigmented patches were observed at the end of differentiation time course (Figure 1A).

In addition to the morphologies and histological findings, the specific features of undifferentiated iPSCs and iPSC-derived lineage cells were validated by examining the biological markers of each cell type. Using immunofluorescence staining, iPSCs exhibiting typical embryonic stem cell-like morphologies were found to be stained strongly positive for Oct4 and Sox2 (Figure 1B). iPSC-MSCs not only presented fibroblast-like morphologies, but were stained positive for CD190 (Figure 1B). The iPSC-RGCs were characterized by the formation of neurites projected from the somas within the optic vesicles. The neurites and somas of iPSC-RGCs were stained positive for beta III-tubulin (Figure 1B). iPSC-RPEs were characterized by an extensive distribution of pigmented patches and positive staining for RPE65 (Figure 1B).

**Figure 2:**
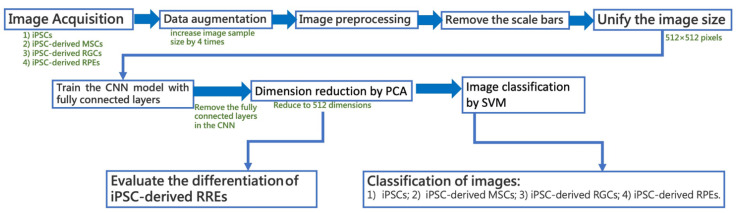
The training scheme of the neural network algorithm for the recognition of cell images.

Briefly, the training of these algorithms for image recognition was conducted as summarized in Figure 2. Overall 1937 images of iPSCs and other differentiated cells were enrolled; including 401 images of iPSCs, 565 images of iPSC-MSCs, 370 images of iPSC-RPEs, and 601 images of iPSC-RGCs. The image file formats were either JPG or TIFF. The magnification of these images was between 10 and 20X. Prior to the training of the CNN, the image dataset was split the training (1291 images) and test (647 images) subsets. The images of the training subset were subjected to data augmentation, which involved horizontal flip, vertical flip, and horizontal and vertical flip combined, resulting in 5164 images in the training subset. 20% (1032 images) of the augmented data were selected as the validation subset. The data in the test subset were not augmented. After the data augmentation, the number of images of iPSCs increased from 401 to 1604, the number of images of iPSC-MSCs increased from 565 to 2260, the number of images of iPSC-RGCs increased from 602 images to 2408, and the number of iPSC-RPE images increased from 370 to 1480. Images were preprocessed to unify their size. To avoid incorrect feature extraction, the scale bars in all figures were removed. Subsequently, we trained the CNN networks which consisted of fully connected layers. After removing the fully connected layers, the PCA was used to reduce the number of dimensions of image deep feature extraction. After the feature projection, SVM was used to classify the pooled images in the validation set into four categories: iPSCs, iPSC-MSCs, iPSC-RGCs, and iPSC-RPEs. The maximal variance between iPSC images and the images to be tested was used to distinguish the features among these four categories. For iPSC-RPEs, the projected distances between iPSC-RPEs and iPSCs were used to evaluate their differentiation degree.

### 2.3. Image Preprocessing

To enhance the capacity of our models to distinguish images at both low and high magnification and minimize the adverse effects of excessive dimensions during the training, images of different cells were preprocessed and assigned for feature scaling, aiming to train the neural network to detect the non-repetitive images during data augmentation. At the macroscopic perspective, we input the 512 × 512 × 3 tensor (macroscopic tensor) into the CNN model, and a copy of this tensor was used as the input of the CNN. At the microscopic perspective, the images were divided equally into four 256 × 256 × 3 tensors in order to detect the subtle differences among images. After the dividing, a macroscopic image and four microscopic images were subjected to five CNNs for the subsequent training. Before the training, the image order was randomly changed to enhance the convergence rate and accuracy, and the random seed was set as zero. Because the input size of the macroscopic tensor was 512 × 512, the average pooling layer was added at the front of the input in the macroscopic tensor to ensure that the inputs were all 256 × 256 × 3 in size in the five CNN models.

### 2.4. Model Design and Training

**Figure 3:**
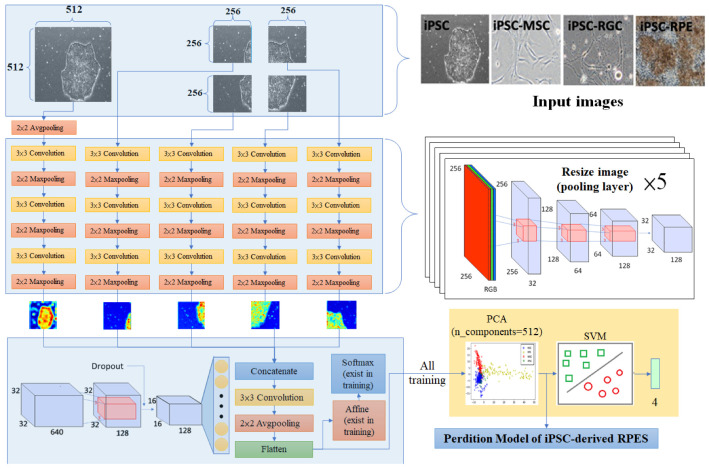
The CNN architecture of the proposed multi-slice tensor model.

Figure 3 illustrates the CNN neural network architecture of the multi-slice tensors shared by all cut images in this study. We subjected all cut images to the CNN that contains three convolutional layers and three maximum pooling layers. After passing through five convolutional layers, the input images were stacked, followed by another convolutional layer and subsequent flattening. After flattening and passing through the fully connected layer, the output results were obtained. The kernel size of these five CNN models was 3 × 3, and the number of filters was 32, 64, and 128 in sequence. After each convolutional layer, a 2 × 2 maximum pooling was performed. Before training, the input images were passed through an average pooling layer of 2 × 2 size, allowing the inputs of the first convolutional layer of the five CNNs to be 256 × 256 × 3 in size. After passing the convolutional layers and the pooling layer, the output 32 × 32 × 128 tensors were obtained. These tensors were then concatenated, merged into 32 × 32 × 640 tensors, and passed through another convolutional layer with 128 filters and a 3 × 3 kernel size. After that, 50% of the neurons were randomly discarded and averagely pooled to prevent overfitting, resulting in the tensors with 16 × 16 × 128 in size. After flattening and passing through a hidden layer containing 128 neurons, the tensors were output to the output layer containing 4 neurons in which the softmax was used as the activation function.

### 2.5. N-Dimensional Classifier Based on the combination of PCA and SVM

SVM is a supervised learning model used for tackling classification problems. Four-dimensional spaces were defined for the target images, i.e., iPSCs, iPSC-MSCs, iPSC-RGCs, and iPSC-RPEs to represent the features of each image. Since SVM may exhibit better performance of classification than the fully connected layer, we calibrated the model after obtaining a certain accuracy by removing the neurons behind the flatten layer and using the flatten layer as an alternative output. After the removal of the flatten layer, the file size was reduced from 61.9 MB to 4.68 MB. All images in the training set were subjected to the calibrated model for further training, generating 5164 arrays with 32,768 dimensions. We then used the MaxAbsScaler in scikit-learn to downsample this array to floating-point numbers between -1 and 1 and subsequently used the machine learning in scikit-learn and PCA to reduce the dimensions of these arrays from 32,768 to 512. Next, we subjected these 5164 arrays with 512 dimensions to the SVM for supervised learning. The addition of PCA and SVM robustly improved the classification effect of the original model. Finally, we modified the feature scaling during preprocessing and changed the input from the range 0 to 1 to −1 to 1, and then retrained the model using the training set.

## 3. Results

### 3.1. Heatmap Visualization of the Image Features

**Figure 4:**
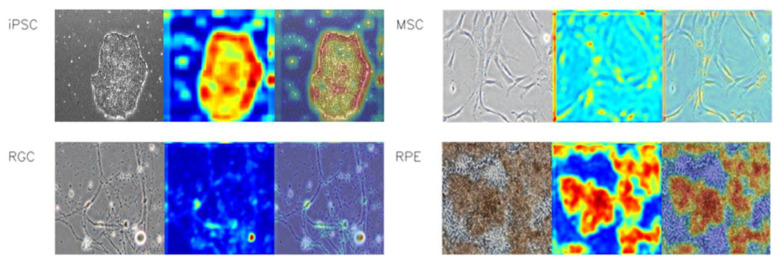
Comparison of four cell types with the heatmap imaging. For each indicated cell type, the left panel shows the representative phase-contrast microscopy image, the middle panel shows the heatmap calculated by the CNN model, and the right panel shows the overlay of the heatmap and the input.

To ensure that the CNN model captured the correct features of the cell images, we used heatmap to visualize the detectable features. iPSCs were characterized by the well-defined borders among all iPSC colonies. In some images, the boundary occupied the entire image. iPSC-MSCs were characterized by the elongated morphologies, which were very subtle on the images. iPSC-RGCs were characterized by extending neurites and iPSC-RPEs were characterized by the dark brown pigmentation. These features could be detected by either macro- or microscopic examination. The features of iPSCs were detectable using macroscopic examination. Considering the subtle features of iPSC-MSCs and iPSC-RGCs, we resized all cell images to a size of 512 × 512 pixels where the subtle features were detectable. All resized images were then subjected to the model and converted into the tensors. After the conversion, the tensors were further cropped, allowing the model to adapt the input images at both macro- and microscopic perspectives. Figure 4 shows the representative features of iPSCs, iPSC-MSCs, iPSC-RGCs, iPSC-RPEs with heatmap visualization.

### 3.2. Scale Bars Confound the Training of CNN

**Figure 5:**
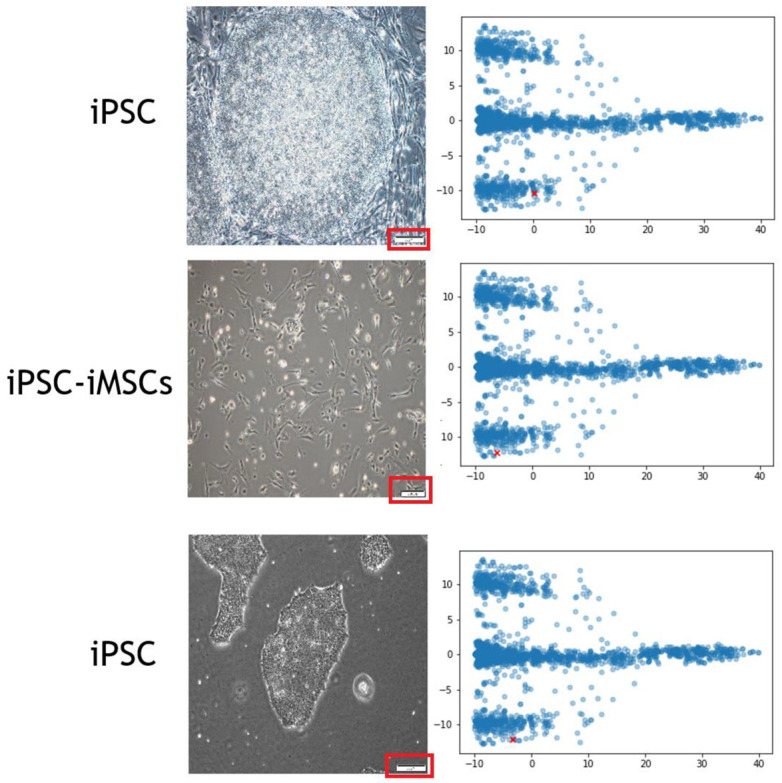
A representative image interpretation by our proposed model. The cell images (left side) with scale bars (red rectangle) were subjected to the training of our proposed model. When using PCA to reduce the dimension (right side), the scale bars can be detected in the feature coordination (red cross point).

When the scale bars were detected at the corner of the images, CNN overemphasized the weights area on the scale bar regions (Figure 5), so we cropped the images in the test set to remove the scale bars during the image preprocessing and subjected them to subsequent training. However, we did not remove the scale bars on the images in the validation set. Comparing to the training in which the images with the scale bars were used, training the CNN using the images without the scale bars have significantly improved the accuracy of image recognition for the images containing the scale bars. In addition, after the dimension reduction using PCA, we were able to easily detect whether the scale bars were included in the images through the feature coordination. This was due to CNN focusing the attention on the scale bars.

### 3.3. Effect of Feature Scaling on the Accuracy and Loss during the Training

**Figure 6:**
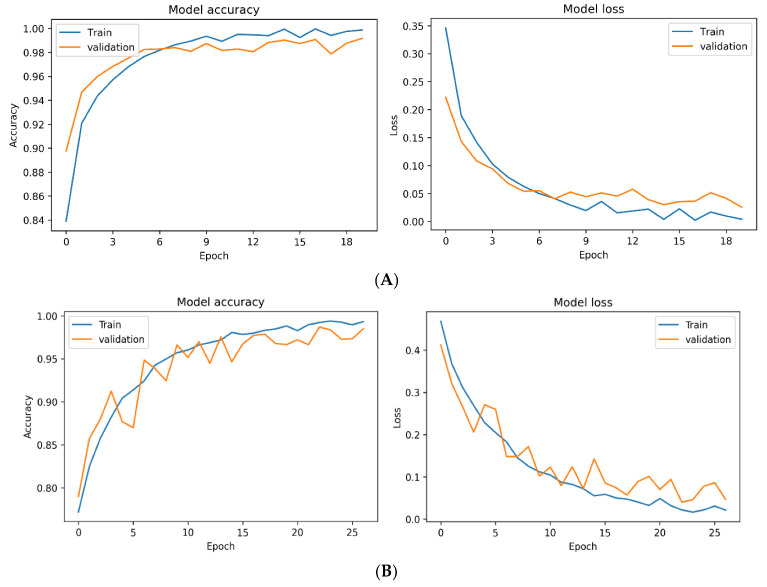
Effect of feature scaling on the accuracy and loss during the training. (**A**) Comparison of training and validation accuracy (**left**) and loss (**right**) with the features between −1 and 1 using accuracy and loss. (**B**) Comparison of training and validation accuracy (**left**) and loss (**right**) with the features between 0 and 1 using accuracy and loss.

We recorded the values of the training process and used Python’s matplotlib library to plot the line charts of model accuracy and model loss. We found that the model exhibited a batch training set accuracy higher than 80% in the first epoch of training, and a steady decline of the loss function (Figure 6A). In addition, when the features were scaled to a range between 0 and 1 instead of −1 to 1 with an average of zero during preprocessing, the training process was relatively unstable with relatively slow training speed (Figure 6B). At the first epoch, the accuracy of the training set was only 80%, significantly less than the previous model trained with an average of zero.

### 3.4. Effect of Image Division on the Performance of the Multi-Slice Tensor Model

The magnifications of all enrolled cell images were between 10 to 20X. To analyze the effect of input images with different magnifications on the verification of the model, we cut each input image into four equal parts. Subsequently, all input images were resized to the size of 512 × 512. The effect of image division in the training set on the performance of the multi-slice tensor model is presented in Table 1.

The difference between the two models was whether the cut input images were included in the training set during training. Invariantly of the presence or absence of the cut input images in the training set, the test set without the cut images exhibited a recognition accuracy of higher than 95%. However, training without the cut input images in the training set showed a recognition accuracy of only 70.6% in the test set containing the cut images. Training the CNN model using the training set that contained the cut input images consistently exhibited a recognition accuracy higher than 97%. Collectively, our findings indicated that expanding the input image data in the training set via image division helped the model to adapt to the recognition of images with different magnification.

In a case of using a single tensor model without recruiting cut input images (only a macroscopic model was used), the resultant accuracy reached 94.4%. However, if the multi-slice tensor model was used, the accuracy increased to 96.5%. When we removed the fully connected layers and used the flatten layer as the input of the SVM to undergo a supervised classification, the accuracy declined to 89.7%. When PCA was added after the flatten layer to reduce the dimensions, which was followed by SVM, the accuracy increased to 97.3%. If we replaced PCA by linear discriminant analysis (LDA) to conduct the supervised dimension reduction, the accuracy declined to 53.9%. We found that this model exhibited good segmentation performance during the training, but poor segmentation performance during the test. Subsequently, we used feature scaling between −1 to 1 as the input to replace the scaling 0 to 1, as a result, the accuracy of 97.2% was obtained after retraining this model. These data indicated that normalization using 0 as the center was beneficial to the model training. When the training was completed, PCA was applied to the flatten layer to conduct the dimension reduction and SVM was used for the supervised classification, as result, the highest accuracy of 97.8% was obtained. The accuracy of different CNN models used in this study is compared and shown in Table 2.

After several training epochs, we were able to accurately predict the 647 images in the test set using our aforementioned training model with an accuracy of 97.8%. Among each classification, the accuracy of iPSCs, iPSC-MSCs, iPSC-RGCs, and iPSC-RPEs prediction were 97%, 97.8%, 97%, and 100%, respectively. We trained this model for 19 epochs with an individual batch size of 8. The confusion matrix summarizing the recognition results is presented in Table 3.

### 3.5. Projection of the Cell Features Using PCA

**Figure 7:**
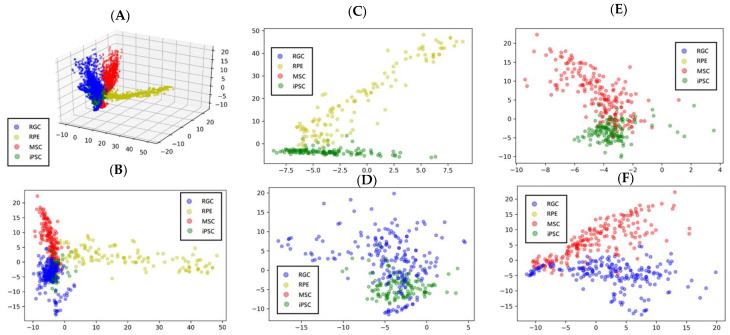
Three-dimensional projection, the maximum projection results, and two-dimensional projection of iPSCs, iPSC-MSCs, iPSC-RPEs, and iPSC-RGCs after using PCA for the output of the multi-slice tensor model. (**A**) The three-dimensional projection of four cell types; (**B**) Results of the maximal 2D projection; (**C**) The projection of iPSCs and iPSC-RPEs (**D**) the projection of iPSCs and iPSC-RGCs; (**E**) the projection of iPSCs and iPSC-MSCs; (**F**) the projection of MSCs and iPSC-RGCs.

Next, we subjected the features extracted by the convolutional layers to a 512-dimension PCA for dimension reduction and feature projection. Notably, the features of iPSC-MSCs, iPSC-RGCs, and iPSC-RPEs were all of higher variability as compared to those of iPSCs (green scatter; Figure 7). When we further measured the coordinates of projections in the feature space, we found that the distance from the spot of any given image to iPSCs could reflect the differentiation status of the image. After dimension reduction using PCA and the evaluation of differentiation degree by detecting the coordinates, we found that the first to third dimensions were the most weighted and could separate these four types of cells in this study. Among these cells, iPSC-RPEs and iPSCs were separable by the value of 0 in the 0th dimension. iPSC-MSCs and iPSCs were separable by the value of 0 in the 1st dimension, and iPSC-RGCs and iPSCs were separable by the value of 0 in the 2nd dimension. In addition, iPSC-RGCs and MSCs were separable by the value of 0 in the 1st dimension. Figure 7 shows the three-dimensional/two-dimensional projection and the maximum projection results of each classification after using PCA for the output of the multi-slice tensor model.

### 3.6. Evaluating the Differentiation Degree of iPSC-RPEs

**Figure 8:**
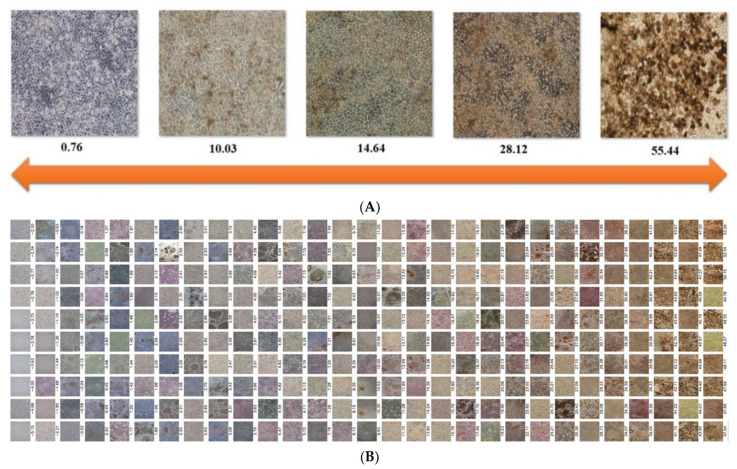
Evaluation of the RPE differentiation degree by the value of the PCA projection. (**A**) From left to right, the larger value reflects higher differentiation degree of iPSC-RPEs. (**B**) Sorting the values of projections of iPSC-RPEs. From left to right: the larger value reflects higher differentiation degree of iPSC-RPEs.

Based upon the scatter plot output by PCA, we found that the 1st dimension showed the best performance to separate the spots of iPSCs and iPSC-RPEs. We took this dimension as an example and renamed each iPSC-RPE image using their coordinates after feature projection and then ordered them accordingly. The iPSC-RPEs with the coordinate further from that of iPSCs exhibited a more obvious dark brown feature of iPSC-RPEs. Figure 8A shows the comparison of the phenotypes and corresponding PCA-projected coordinates of each iPSC-RPE image. iPSC-RPEs with optimal differentiation may represent ideal cell source for clinical transplantation. Figure 8B shows the sorting of the values of projections of iPSC-RPEs to represent the differentiation degree.

**Figure 9:**
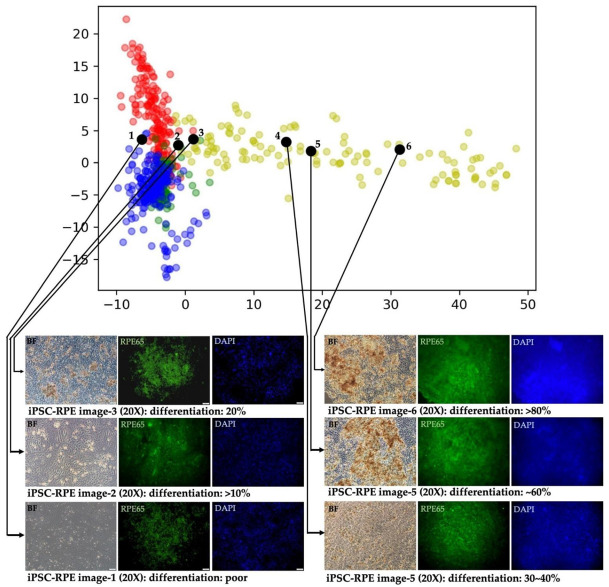
Using iPSC-RPEs with various differentiation degree as the input to verify the accuracy of iPSC-RPE image recognition by the multi-slice tensor model. In the scatter plot of iPSC-RPEs (yellow green spots), iPSC-RPEs with poor differentiation were projected to the left side, iPSC-RPEs with moderate differentiation to the middle, and iPSC-RPEs with high differentiation-to the right side of the scatter plot.

To further verify the accuracy of iPSC-RPE image recognition by this proposed model, we picked up 6 dishes of iPSC-RPEs with various differentiation degree after undergoing the complete differentiation time course (Figure 9) using protocols reported by either Regent et al. [28] or Yang et al. [8] The differentiation degree of each iPSC-RPE dish was interpreted by qualified technicians after microscopic examination. Subsequently, the images of these cells were used as the input in the multi-slice tensor model and output using PCA. These iPSC-RPEs with various differentiation degree were also subjected to immunofluorescence staining and the RPE65 staining of each dish of iPSC-RPE was obtained. As shown in Figure 9, the iPSC-RPEs with poor differentiation degree, as validated by low RPE65 staining, were projected to the left side of the iPSC-RPE scatter plot. iPSC-RPEs with 40–60% differentiation degree were projected to the middle part of the iPSC-RPE scatter plot. iPSC-RPEs with optimal differentiation degree (more than 80%) were projected to the right side of the iPSC-RPE scatter plot. Collectively, our data demonstrated that our proposed CNN model could be used for evaluating the differentiation degree of iPSC-derived cell lineages.

## 4. Discussion

In this study, we developed a multi-slice tensor model accompanied by dimension reduction by PCA and supervised classification by SVM to screen iPSC-derived cell lineages with an accuracy of 97.8%, When we added the PCA after the flatten layer of this model to measure the coordinates of projections in the feature space, the differentiation degree of iPSC-RPEs was accurately recognized. We demonstrated the potential of this proposed multi-slice tensor model as a tool for efficient and reliable biological image recognition for the selection of iPSC-RPEs after defined iPSC-RPE differentiation. Overall, this multi-slice tensor model is able to distinguish differentiated cells of different cell lineages with corresponding unique features and help the selection of iPSC-RPEs with optimal differentiation.

The major advances in the clinical use of iPSC-based technologies in cell therapy have been made possible due to the fact that iPSCs represent an infinite source of any type of human cells. iPSCs can be obtained from reprogramming an adult somatic cell and are capable of proliferation, differentiation, and replacement of damaged cells, enabling their dynamic niche in regenerative medicine. To date, there is a broad range of ongoing research in cell therapies for various diseases using iPSCs-derived cell types ranging from dopaminergic progenitors for Parkinson’s disease [30], iPSC-derived cardiac progenitors for heart failure [31], iPSC-derived beta-pancreatic cells for type I diabetes [32], iPSC-derived NK cells for advanced solid tumors [33], and iPSC-RPEs for retinal disorders [16,34]. Stemming from these recent advances, there are growing expectations toward quality control in the mass production of iPSCs for their respective clinical applications.

Effective quality control of stem cells largely determines the success of stem cell therapy. However, the analysis of iPSC-derived colonies requires manual identification, which is time-consuming, labor-intensive, and error-prone. Hence, before human iPSCs can be applied as a standard method and manufactured in large scale, the decision-making must be automated to ensure an efficient and objective quality control process. AI, an emerging field of computer science and engineering, has shown potential applications in stem cell research, including understanding the behavior of iPSCs, recognizing individual iPSC-derived cell types, and the characterization and evaluation of cell qualities in transplantations [35,36]. Machine learning-based AI algorithms have become a standard method for various aspects of image analysis, and, in particular, in the recognition and analysis of iPSC colony images [25,37,38]. Such algorithmic frameworks range from the convolutional neural networks (CNNs) to the more traditional algorithms, such as support vector machines (SVMs), principal component analysis (PCA), random forests (RFs), decision trees (DTs), and multi-linear perceptions (MLPs). To date, a limited number of studies describes the application of CNNs for clinical stem cell analysis, particularly related to iPSCs. These studies delineate the potential application of CNNs for the analysis of iPSC microscopy images [39], the identification of iPSC-derived cell types, such as endothelial cells based only on their morphology [40], and the identification of very early onset pluripotent stem cells differentiation [41]. More traditional algorithms have also been used in the studies concerning the image recognition of stem cells too. Kavitha et al., have shown that the SVMs, RFs, and AdaBoost algorithms are able to recognize and determine the health of iPSCs better than the DTs and MLPs, although all five learning methods achieved accuracies of more than 87.5% [42]. In a separate study, they also demonstrated that the CNN-based approach achieved an accuracy of 93.2% in classifying iPSCs compared to 83.4% by the SVM model [43]. One issue for improving the accuracy of SVMs is finding an appropriate kernel for the given data. Joutsijoki et al., showed that the intensity histograms-based SVM using linear kernel functions alone is not adequate for iPSC colony image classification, achieving the accuracy of only 54% [44]. Wakui et al., used a non-linear SVM classifier to assess the quality of three types of iPSC cells and obtained an average accuracy of more than 80%. [45] Zanaty et al., introduced a new kernel function that can be used for both linear and non-linear datasets to improve the classification accuracy of SVMs, called the Gaussian radial basis polynomials function (GRPF). The GRPF-based SVM approach achieved an average accuracy of 95.79% that was far greater than the average accuracy of 85.77% in the RBF-based kernel SLM and 84.90% in the MLP [46]. Additionally, there have also been studies that used a combination of CNN and SVM to improve the image recognition accuracy. Acevedo et al., first used CNN as a tool for feature extraction, and then used SVM to use these features for classification. The accuracy achieved was as high as 96 and 95%, compared to the accuracies of 86% and 90% obtained when only the CNN models VGG16 and SingingV3 were used, respectively [47]. Cascio et al., also adopted a similar approach to classify indirect immunofluorescence images, achieving an accuracy of 96.4%. Features were extracted using an AlexNet-based network and then, cell pattern association was performed using SVMs and k-nearest neighbors (KNN) classifier [48].

So far, there are still several drawbacks in our proposed multi-slice tensor model. The model was trained by the input using the images containing single cell type and was able to distinguish iPSCs, iPSC-MSCs, iPSC-RPEs, and iPSC-RGCs in the validation set with high accuracy. However, it remains unclear whether this multi-slice tensor model is able to simultaneously recognize different iPSC-derived lineage cells in an image containing the co-culture of different lineage cells. This model did not ensure the selection of high quality iPSCs and exclusion of bad quality iPSCs. In addition, although this model showed remarkable performance in recognizing the differentiation degree of iPSC-RPEs, our findings did not confirm that this model could be used for the evaluation of differentiation degree in iPSC-MSCs and iPSC-RGCs. In addition to the formation of fibroblast-like phenotypes, iPSC-MSCs did not present other unique and visualizable features after the end of differentiation. Our model recognized iPSC-RGCs by detecting the extensive formation of neurites. Nevertheless, the optic vesicles, form which the neurites projected, were heterogeneously consisted of somas and other cell structures (Figure 1). On the other hand, can be individually separated manually, however, it requires significant manpower [9,10]. The optic nerve is well known not to regenerate, and the repair of damaged optic nerves using iPSC-RGCs remains a hurdle in clinical transplantation [49].

## 5. Conclusions

In this study, we used a combination of multi-slice tensor model and PCA to reduce dimension for feature extraction, and SVM and CNN to recognize and classify iPSC colony images. This is the first of such studies among stem cell studies to the best of our knowledge. Our model achieved a high accuracy in distinguishing between the iPSCs and iPSC-derived MSCs, RGCs, and RPEs. More notably, the model was able to distinguish the quality and extent of maturation of iPSC-derived RPEs, which may serve as an important tool in the ongoing efforts of pre-clinical studies and clinical transplantations of iPSC-derived RPEs [16,34]. If only the single tensor model was used without recruiting cut input images, the accuracy reached 94.4%. If the multi-slice tensor model was used, the accuracy reached 96.5%. When the fully connected layers were removed and only the flatten layer was used as the SVM input, the accuracy decreased to 89.7%. When PCA was used upon the flatten layer for SVM input, the accuracy increased to 97.3%. The hybrid methodology of PCA + SVM and promising results herein demonstrate potential in automating the interpretation and early detection of stem cells, thereby assisting researchers in the stem cell culture progress and making a big step forward in the development of regenerative and precision medicine. Expectably, owing to the abilities of this model to accurately recognize well-differentiated iPSC-RPEs, it is possible to employ this multi-slice tensor model to select the candidate cells with ideal features, and simultaneously exclude cells with immature/abnormal phenotypes. After some fine-tuning and optimization, this rapid screening system may hold promises and facilitate the manufacturing of clinical grade iPSC-RPEs and help their translation into clinical cell therapy. Further preclinical studies and validation is still required to verify the applicability of this system.

## Figures and Tables

**Table 1:** Effect of image division in the training set on the accuracy of the multi-slice tensor model.

Training Set	Test Set	
	With Cut Images	Without Cut Images
With cut images	97.2%	97.9%
Without cut images	70.6%	96.4%

**Table 2:** The comparison of CNN models.

Input	CNN Model	Classification	Accuracy
0 to 1	Single tensor	fully connected layers	94.4%
0 to 1	multi-slice tensors	fully connected layers	96.5%
0 to 1	multi-slice tensors	SVM	89.7%
0 to 1	multi-slice tensors	PCA + SVM	97.3%
0 to 1	multi-slice tensors	LDA + SVM	53.9%
−1 to 1	multi-slice tensors	fully connected layers	97.2%
−1 to 1	multi-slice tensors	PCA + SVM	97.8%

**Table 3:** Confusion matrix summarizing the recognition results by the multi-slice tensor model.

Average Accuracy: 97.8%	Recognition				Recall	F Score
	iPSC	iPSC-MSC	iPSC-RGC	iPSC-RPE		
iPSC	130	1	2	1	97.0%	97.4%
iPSC-MSC	0	185	3	1	97.8%	97.8%
iPSC-RGC	1	5	195	0	97.0%	97.4%
iPSC-RPE	0	0	0	123	100%	98.9%

## Data Availability

Not applicable.
